# Impact of tirzepatide on systemic arterial stiffness assessed by cardio-ankle vascular index in individuals with obesity complicated by type 2 diabetes: A retrospective cohort study in Japan

**DOI:** 10.1016/j.obpill.2026.100291

**Published:** 2026-06-24

**Authors:** Daiji Nagayama, Miwako Wakamatsu, Yasuhiro Watanabe, Maika Ikeda, Yuta Koshikawa, Osamu Horikawa, Sayaka Tsuji, Kohji Shirai, Atsuhito Saiki

**Affiliations:** a Department of Internal Medicine, Nagayama Clinic, Oyama-City, Tochigi, 323-0032, Japan; b Center of Diabetes, Endocrinology and Metabolism, Toho University, Sakura Medical Center, Sakura-City, Chiba, 285-0841, Japan; c Department of Internal Medicine, Mihama Hospital, Chiba-City, Chiba, 261-0013, Japan

**Keywords:** Body composition, Cardio-ankle vascular index, Obesity, Tirzepatide, Type 2 diabetes, Vascular function

## Abstract

**Background:**

Tirzepatide, a multi-agonist incretin agent that targets body weight and metabolic parameters, has stronger weight reduction and glycemic control effects than existing agents. This study aimed to elucidate the effects of tirzepatide on vascular function in individuals with obesity complicated by type 2 diabetes (T2D).

**Methods:**

This retrospective observational cohort study evaluated the effects of weekly tirzepatide (5–15 mg, median treatment duration 12.7 months) on vascular function and body composition in 24 individuals [45.8% male, median 48.6 years, body mass index (BMI) 32.3 kg/m^2^] with obesity and T2D. Sixteen individuals (66.7%) were switched from glucagon-like peptide-1 receptor agonists (GLP-1 RAs). Vascular function was assessed using the cardio-ankle vascular index (CAVI), and body composition was evaluated using skeletal muscle index (SMI) and body fat index (BFI) measured by InBody720.

**Results:**

Tirzepatide treatment significantly decreased CAVI (post-treatment to baseline: median 8.5 to 7.8) accompanied by significant reductions in BMI (mean 33.8 to 31.8 kg/m^2^), SMI (median 11.4 to 10.7 kg/m^2^), BFI (median 12.8 to 11.3 kg/m^2^) and glycemic parameters (including HbA1c: median 6.6 to 5.6%). The urinary albumin/creatinine ratio (UACR) tended to decrease with tirzepatide (median 11.9 to 7.2 mg/gCr, *p* = 0.067), and change in (Δ)UACR showed a trend of positive correlation with ΔCAVI (*r*_*s*_ = 0.403, *p* = 0.057). BMI reduction was greater (*p* = 0.040) and CAVI reduction tended to be greater (*p* = 0.089) at higher tirzepatide doses (10 and 15 mg) than at lower doses (5 and 7.5 mg). Prior GLP-1 RA use did not affect tirzepatide efficacy. Baseline SMI, but not BFI, correlated significantly and negatively with ΔCAVI (*r*_*s*_ = −0.424).

**Conclusion:**

Tirzepatide may dose-dependently reduce body weight and CAVI, contributed by decreased UACR and relatively high baseline SMI. Although tirzepatide is expected to improve kidney and vascular dysfunction in individuals with obesity complicated by T2D, careful attention to body composition is warranted.

## Graphical abstract

**Figure:**
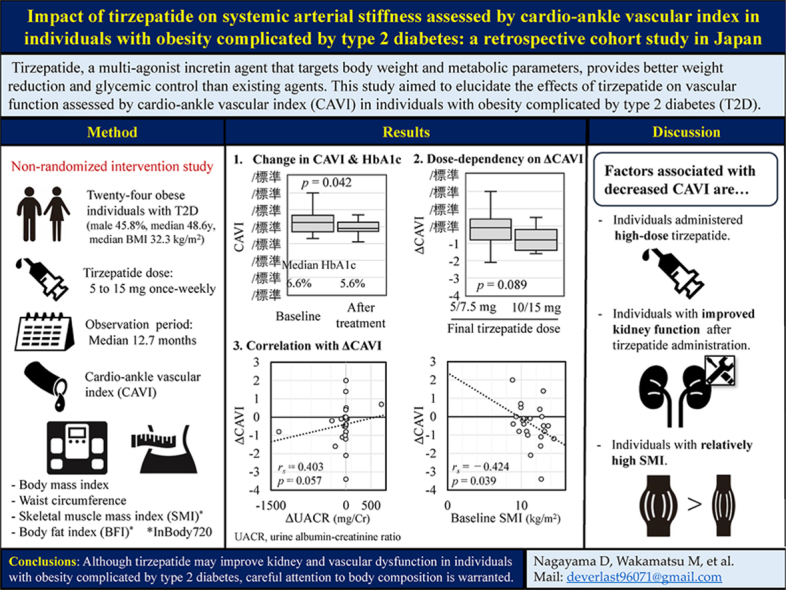

## 1 Introduction

Tirzepatide, a dual agonist of glucose-dependent insulinotropic polypeptide (GIP) and glucagon-like peptide-1 (GLP-1) receptors, is a novel agent that is transforming treatment for obesity-related complications. It provides superior glycemic control compared to contemporary anti-type 2 diabetic agents, including glucagon-like peptide-1 receptor agonists (GLP-1 RAs) [1]. Furthermore, study has indicated that tirzepatide contributes to prevent cardiovascular disease (CVD) not only through its blood glucose-lowering effects but also by improving abdominal obesity [2]. In addition, tirzepatide has been hypothesized to exert direct renoprotective effects beyond its metabolic actions, and novel findings have emerged suggesting potential benefits in protecting against progression of chronic kidney disease (CKD) [3]. However, the detailed effects of tirzepatide on vascular function have not yet been confirmed.

Cardio-ankle vascular index (CAVI) is a non-invasive index of systemic arterial stiffness, which is calculated from the heart–ankle pulse wave velocity and blood pressure (BP) based on the stiffness parameter β, and is designed to be relatively independent of acute BP fluctuations at the time of measurement [4]. CAVI reflects the stiffness of the aorta, femoral and tibial arteries, thereby providing an integrated assessment of central-to-peripheral arterial properties across a wide arterial tree. Previous clinical studies have demonstrated that increased CAVI is associated with the prevalence and incidence of CVD including coronary artery disease, stroke and heart failure, suggesting that CAVI may serve as a useful marker for cardiovascular (CV) risk stratification [5]. Interventional studies have provided further evidence that CAVI can be decreased by various therapeutic strategies, such as antihypertensive agents, lipid-lowering therapy, glucose-lowering drugs and lifestyle modification, indicating that CAVI is a modifiable vascular biomarker that may reflect the effectiveness of CV risk management.

Diabetes mellitus is one of the most clinically important complications with obesity, and this metabolic disorder is independently associated with increased CAVI [6]. However, reduction of glycated hemoglobin (HbA1c) alone does not necessarily improve vascular dysfunction. Instead, therapeutic interventions that mitigate postprandial hyperglycemia [7,8], ameliorate insulin resistance and reduce oxidative stress [9,10] have been reported to achieve meaningful decrease in CAVI. However, major clinical questions regarding tirzepatide remain unsolved, such as how the drug decreases CAVI, and, if beneficial, through what mechanism. If tirzepatide is confirmed to improve vascular function, its clinical relevance may extend beyond diabetes and obesity control, but also as an anti-atherosclerotic agent.

We hypothesized that tirzepatide treatment improves vascular function as indicated by CAVI in individuals with obesity and type 2 diabetes. To test this hypothesis, we designed a retrospective study to analyze the effect of tirzepatide on CAVI in these individuals, and explore the relationship between tirzepatide doses and CAVI changes as well as factors associated with CAVI changes.

## 2 Materials and methods

### 2.1 Study population and design

This was a retrospective observational cohort study evaluating the effects of once-weekly tirzepatide on vascular function and body composition, and conducted at the Center of Diabetes, Endocrinology and Metabolism, Toho University Sakura Medical Center, Sakura, Chiba, Japan, operating under the Japanese national health insurance system. The inclusion criteria for this study were individuals with a BMI of at least 27 kg/m^2^ who had diabetes in combination with hypertension and/or dyslipidemia, or those with a BMI of at least 35 kg/m^2^ who had diabetes alone. Immediately after being diagnosed with diabetes, the individuals received counseling and guidance on diet and exercise. If adequate diabetes control was not achieved after more than three months, treatment with established antidiabetic agents was initiated. When those treatments failed to adequately control the severity of diabetes and/or obesity-related complications after more than six months, treatment was switched to tirzepatide. If they were receiving dipeptidyl peptidase (DPP)-4 inhibitors or GLP-1 RAs, those medications were discontinued. Individuals were initially treated with once-weekly injection of 2.5 mg of tirzepatide. Four weeks later, if no adverse effects were observed and proper injection technique was confirmed, the dose of tirzepatide was increased to 5 mg. Subsequently, the dose was increased in 2.5 mg increments as needed, and some participants reached a maximum dose of 15 mg. Individuals who had received tirzepatide for 12 months or longer were retrospectively enrolled in this study. Participants underwent two evaluations of vascular function and body composition using CAVI and the InBody720 device (described below), respectively. The first evaluation was conducted no later than 3 months prior to the initiation of tirzepatide, and the second 9–15 months after initiation. Individuals with a history of severe hepatic dysfunction, CVD and/or peripheral arterial disease were excluded from this study. Finally, a total of 24 Japanese individuals with obesity complicated by type 2 diabetes who started tirzepatide treatment between June 2023 and February 2025 were enrolled in this study.

### 2.2 Data collection

All physical and laboratory parameters were measured and assessed by standardized methods. Height and weight were measured, and body mass index (BMI) was calculated as weight (kg) divided by height squared (m^2^). Waist circumference (WC) was measured horizontally at the height of the umbilicus, with the individual standing with arms hanging relaxed. Blood pressure (BP) was measured from an upper arm cuff after resting for 5 min in a sitting position. Hypertension was defined as either systolic BP (SBP) ≥ 140 mmHg and diastolic BP (DBP) ≥ 90 mmHg, or treatment with BP-lowering agents. Blood was collected from an anterior upper extremity vein in the morning after a 12-h fast for measuring fasting plasma glucose (FPG, mg/dL), total cholesterol (TC, mg/dL), triglycerides (TG, mg/dL) and high-density lipoprotein cholesterol (HDL-C, mg/dL). Type 2 diabetes mellitus was diagnosed based on the World Health Organization (WHO, Geneva, Switzerland) and American Diabetes Association (ADA, Arlington, Virginia, USA) criteria [11]. Low-density lipoprotein cholesterol (LDL-C, mg/dL) was calculated using the Friedewald formula: LDL-C = (TC) − (HDL-C) − (TG/5). Dyslipidemia was defined as TC ≥ 220 mg/dL, HDL-C < 40 mg/dL, and/or TG ≥ 150 mg/dL, or treatment with lipid-lowering drugs. Estimated glomerular filtration rate (eGFR) was calculated using the following formula developed by the Japanese Society of Nephrology: eGFR (mL/min per 1.73 m^2^) = 194 × Creatinine (mg/dL)^−1.094^ × Age (years)^−0.287^ ( × 0.739 if female). Urinary albumin-to-creatinine ratio (UACR) in spot urine samples collected in the morning was measured using an immunoturbidimetric assay. Current smoking and habitual alcohol consumption were determined using a questionnaire. Alcohol consumption at least once a week was defined as habitual drinking. Skeletal muscle mass and body fat mass were measured using a multifrequency bioelectrical impedance analyzer (InBody720, InBody Co., Ltd., Seoul, Korea). The analyzer uses segmental multifrequency bioelectrical impedance to estimate body composition, and all procedures followed the manufacturer's recommended protocol. Skeletal muscle index (SMI) and body fat index (BFI) were calculated by dividing skeletal muscle mass or body fat mass by height squared (m^2^).

### 2.3 Measurement of arterial stiffness parameters and blood pressure

CAVI was measured using an oscillometric device (VaSera, Fukuda Denshi Co., Tokyo, Japan). With the participant resting in supine position for at least 5 min, cuffs were wrapped around both arms and ankles, and electrocardiographic electrodes together with a phonocardiographic microphone were placed on the chest. The device simultaneously recorded pulse waveforms and brachial blood pressure and automatically calculated CAVI from heart-ankle pulse wave velocity and BP using a previously described algorithm [4].

### 2.4 Statistical analysis

The SPSS software (version 27.0.1, Chicago, IL, USA) was used for statistical analyses. Data were expressed as median (interquartile range [IQR]), and paired groups were compared using the Wilcoxon signed-rank test. However, when trends of the median (IQR) were difficult to interpret using this nonparametric test, paired *t*-test was also performed after confirming normality using the Shapiro–Wilk test, and the results were expressed as mean ± standard deviation (SD). Mann-Whitney *U* test was performed to examine differences between two independent groups. Spearman's rank correlation coefficient (*r*_*s*_) was calculated to assess the relationship between CAVI or change in (Δ)CAVI and clinical variables. In all comparisons, two-sided *p* values less than 0.05 were considered statistically significant. Although not statistically significant, *p* values > 0.05 and <0.10 were regarded as suggestive of a trend.

## 3 Results

### 3.1 Participant characteristics at baseline and after tirzepatide treatment

Twenty-four Japanese individuals (male: female = 11 : 13; baseline median age 48.6 years) with obesity complicated by type 2 diabetes were enrolled in this study (Table 1). The median (IQR) duration of diabetes was 11 (4, 14) years, and median observation period was 12.7 months. At baseline, 50.0% of the individuals had hypertension and 37.5% had dyslipidemia (concurrently in some). Before initiation of tirzepatide, 16 individuals (66.7%) were receiving semaglutide (weekly injection: 50.0%, daily oral administration: 16.7%). Participants who were receiving GLP-1 RAs or DPP-4 inhibitors discontinued those medications upon switching to tirzepatide.

**Table 1:** Participant characteristics at baseline and after tirzepatide treatment.

Variables	Baseline		After treatment		*p* value
	Median	IQR	Median	IQR	
Number (Male/Female)	11/13	–	–	–	–
Age (years)	48.6	(43.7, 56.3)	51.5	(45.0, 58.0)	<0.001
Duration of diabetes (years)	11	(4, 14)	12	(5, 15)	<0.001
Height (meter)	1.68	(1.58, 1.71)	–	–	–
Weight (kg)	91.4	(77.0, 103.6)	88.3	(70.6, 100.1)	0.009
BMI (kg/m^2^)	32.3	(29.6, 36.5)	32.8	(28.4, 34.5)	0.008
	33.8 ± 6.0		31.8 ± 5.2		0.005*
WC (meter)	1.10	(1.02, 1.20)	1.08	(0.97, 1.16)	0.006
Skeletal muscle mass (kg)	28.8	(25.4, 37.7)	29.4	(23.9, 36.3)	0.002
Body fat mass (kg)	36.3	(26.3, 40.3)	32.3	(25.3, 40.3)	0.025
SMI (kg/m^2^)	11.0	(10.1, 12.7)	10.7	(10.0, 12.3)	0.002
BFI (kg/m^2^)	12.8	(10.0, 16.5)	11.3	(9.2, 15.2)	0.026
SBP (mmHg)	135	(116, 142)	134	(124, 140)	0.770
DBP (mmHg)	85	(75, 92)	88	(82, 94)	0.286
FPG (mg/dL)	106	(98, 134)	98	(88, 114)	0.038
HbA1c (%)	6.6	(5.6, 7.1)	5.6	(5.2, 5.9)	<0.001
TC (mg/dL)	191	(171, 229)	195	(172, 231)	0.909
HDL-C (mg/dL)	59	(51, 74)	62	(49, 72)	0.670
TG (mg/dL)	111	(73, 154)	98	(70, 164)	0.354
LDL-C (mg/dL)	112	(86, 133)	111	(82, 137)	0.903
Creatinine (mg/dL)	0.77	(0.62, 0.91)	0.77	(0.61, 0.94)	0.503
eGFR (mL/min/1.73m^2^)	82.3	(53.5, 93.4)	78.6	(52.6, 93.4)	0.346
UACR (mg/gCr)	11.9	(5.3, 75.5)	7.2	(4.0, 30.9)	0.067
Drug received at baseline (% of individuals)					
- Metformin (%)	12.5	–	–	–	–
- Thiazolidine (%)	0.0	–	–	–	–
- Alpha-glucosidase inhibitors (%)	0.0	–	–	–	–
- Sulfonylureas	4.2	–	–	–	–
- Imeglimin	0.0	–	–	–	–
- SGLT2 inhibitors	45.8	–	–	–	–
- DPP4 inhibitors	4.2	–	–	–	–
- GLP-1 RAs	66.7	–	–	–	–
- Insulin	8.3				
- Antihypertensive medicines	50.0	–	–	–	–
- Lipid-lowering agents	37.5	–	–	–	–
Habitual alcohol drinking (%)	37.5	–	–	–	–
Current smoking (%)	45.8	–	–	–	–

The median observation period was 12.7 months. Data are presented as median (IQR) and analyzed by Wilcoxon signed rank-test, or *mean ± SD and analyzed by paired *t*-test. GLP-1 RAs or DPP-4 inhibitors were discontinued when tirzepatide treatment was initiated. BMI, body mass index; WC, waist circumference; SMI, skeletal muscle index, BFI, body fat index; SBP, systolic blood pressure, DBP, diastolic blood pressure; FPG, fasting plasma glucose, HbA1c, glycated hemoglobin; TC, total cholesterol; HDL-C, high-density lipoprotein cholesterol, TG, triglyceride, LDL-C, low-density lipoprotein cholesterol; eGFR, estimated glomerular filtration rate; UACR: urine albumin–creatinine ratio; SGLT2 inhibitors, sodium-glucose transport 2 inhibitors; DPP4 inhibitors, dipeptidyl peptidase inhibitors; GLP-1 RA, glucagon-like peptide-1 receptor agonists; IQR, interquartile range; SD, standard deviation.

**Fig. 1:**
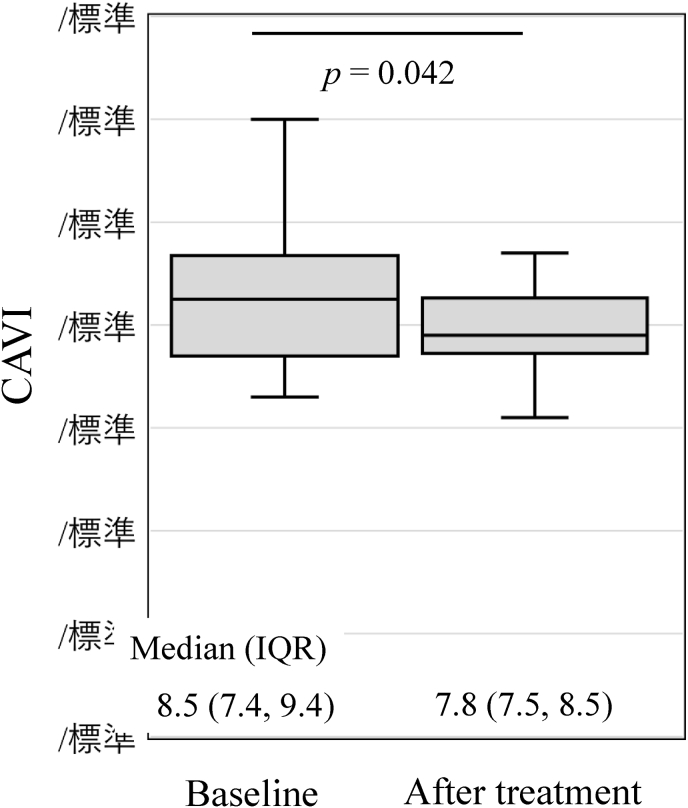
**Change in CAVI before and after tirzepatide treatment.** The median observation period was 12.7 months. Data were analyzed by Wilcoxon signed-rank test. Bottom of the box is 25th percentile, top is 75th percentile, and horizontal line is median. Whiskers represent 10th and 90th percentiles. CAVI, cardio-ankle vascular index; IQR, interquartile range.

After tirzepatide treatment, significant decreases in weight, WC, SMI, BFI, FPG and HbA1c were observed (Table 1). Although a shift in the distribution of BMI (increase in median and decrease in IQR) was observed, the mean decreased by 5.9% (33.8–31.8 kg/m^2^). The UACR showed a trend toward decrease from a median of 11.9 to 7.2 mg/gCr, with borderline statistical significance (*p* = 0.067). The median CAVI decreased significantly from 8.5 to 7.8 (*p* = 0.042), as shown in Fig. 1.

### 3.2 Relationship of final tirzepatide dose or prior GLP-1RA use with change in CAVI

**Fig. 2:**
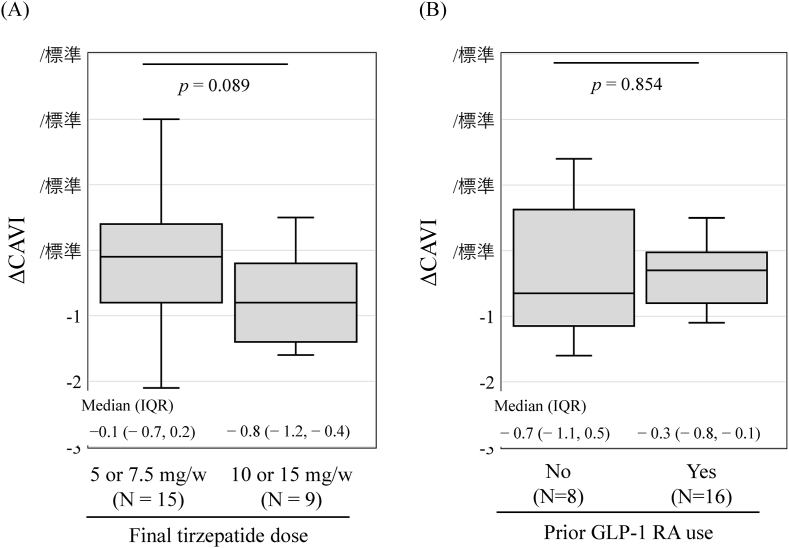
**Relationship of (A) final tirzepatide dose with ΔCAVI and (B) prior GLP-1 RA use with** Δ**CAVI.** Data were analyzed by Mann-Whitney *U* test. Bottom of the box is 25th percentile, and top is 75th percentile, and horizonal line is median. Whiskers represent 10th and 90th percentiles. Δ; change in; CAVI, cardio-ankle vascular index; GLP-1 RA, glucagon-like peptide-1 receptor agonist; IQR, interquartile range.

Fig. 2 shows the relationship of the final tirzepatide dose or prior GLP-1RA use with ΔCAVI. The usual maintenance doses of tirzepatide range from 5 mg to 15 mg once-weekly. Among the 24 individuals in this study, the final tirzepatide dose was 5 mg in 7 individuals (29.2%), 7.5 mg in 8 (33.3%), 10 mg in 6 (25.0%) and 15 mg in 3 (12.5%). We conveniently classified the individuals who received 5 mg or 7.5 mg into a low-dose group and those who received 10 mg or 15 mg into a high-dose group, and compared the changes in CAVI between the two groups. As shown in Fig. 2A, CAVI reduction tended to be greater in the high-dose group compared to the low-dose group, although the difference did not reach statistical significance (*p* = 0.089). Additionally, BMI showed a greater decrease in the high-dose group than in the low-dose group [median (IQR): −4.2 (−6.1, −1.5) vs. −1.6 (−2.5, 1.0), *p* = 0.040].

We also compared ΔCAVI between individuals who were receiving GLP-1 RAs before switching to tirzepatide and those who were not. Of the 24 individuals, 4 (16.7%) received daily oral semaglutide (7 mg in 1, 14 mg in 3) and 12 (50.0%) received once-weekly semaglutide injection (0.5 mg in 3, 1 mg in 8, and 2 mg in 1). All these 16 individuals were switched to once-weekly tirzepatide injection at the start of this study. The efficacy of tirzepatide was compared between 16 individuals (66.7%) who had prior semaglutide treatment and 8 (33.3%) who had not. No significant difference in ΔCAVI was observed between the two groups (Fig. 2B). Additionally, no significant difference in ΔBMI was observed (*p* = 0.806).

### 3.3 Simple regression analysis of correlation between change in CAVI and clinical variables

Next, we examined the clinical variables associated with ΔCAVI after tirzepatide treatment, as shown in Table 2. Simple regression analysis revealed that ΔCAVI correlated positively with ΔTC, ΔTG and Δ LDL-C. In addition, a trend toward a significant positive correlation was observed between ΔCAVI and ΔUACR (*r*_*s*_ = 0.403, *p* = 0.057), although the relationship did not reach statistical significance. On the other hand, changes in anthropometric indices including ΔBMI, ΔSMI and ΔBFI did not correlate with ΔCAVI.

**Table 2:** Simple regression analysis of the correlation between change in CAVI and changes in clinical variables.

ΔCAVI vs.	*r*_*s*_	*p* value
ΔBMI (kg/m^2^)	0.218	0.307
ΔWC (m)	0.212	0.331
ΔSMI (kg/m^2^)	−0.086	0.688
ΔBFI (kg/m^2^)	0.175	0.414
ΔSBP (mmHg)	0.129	0.548
ΔDBP (mmHg)	0.333	0.112
ΔFPG (mg/dL)	0.102	0.636
ΔHbA1c (mg/dL)	0.303	0.149
ΔTC (mg/dL)	0.543	0.006
ΔHDL-C (mg/dL)	0.053	0.805
ΔTG (mg/dL)	0.508	0.011
ΔLDL-C (mg/dL)	0.545	0.006
ΔCreatinine (mg/dL)	−0.227	0.287
ΔeGFR (ml/min/1.73m^2^)	0.118	0.584
ΔUACR (mg/gCr)	0.403	0.057

*r*_*s*_, Spearman's rank correlation coefficient; Δ, change in; CAVI, cardio-ankle vascular index; BMI, body mass index; WC, waist circumference; SMI, skeletal muscle index, BFI, body fat index; SBP, systolic blood pressure, DBP, diastolic blood pressure; FPG, fasting plasma glucose, HbA1c, glycated hemoglobin; TC, total cholesterol; HDL-C, high-density lipoprotein cholesterol, TG, triglyceride, LDL-C, low-density lipoprotein cholesterol; eGFR, estimated glomerular filtration rate; UACR: urine albumin–creatinine ratio.

### 3.4 Relationship of baseline body composition with change in CAVI

**Fig. 3:**
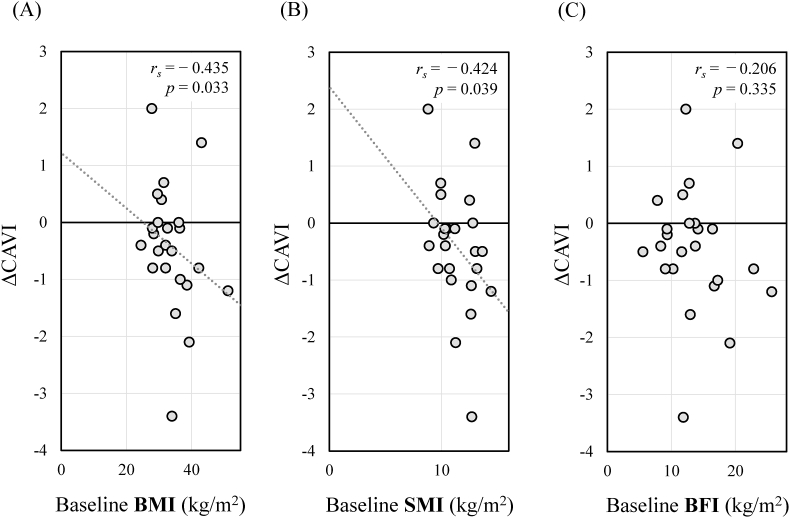
**Relationship of baseline (A) BMI, (B) SMI or (C) BFI with ΔCAVI.***r*_*s*_, Spearman's rank correlation coefficients; Δ; change in; CAVI, cardio-ankle vascular index; BMI, body mass index; SMI, skeletal muscle index; BFI, body fat index.

Finally, to identify the anthropometric indices affecting ΔCAVI, the correlation of baseline BMI, SMI or BFI with ΔCAVI was examined, as shown in Fig. 3. Baseline BMI and SMI correlated negatively with ΔCAVI, whereas baseline BFI did not. On the other hand, baseline SMI did not correlate with ΔSMI (*r*_*s*_ = −0.227, *p* = 0.286, data not shown), suggesting that ΔSMI after tirzepatide treatment was independent of the baseline value.

## 4 Discussion

The American Diabetes Association (ADA) Standards of Care 2025 [12] support the use of incretin-based agents with high weight reduction efficacy, such as tirzepatide, particularly in individuals with obesity complicated by type 2 diabetes. In accordance with this policy, the present retrospective cohort study focused on the management of obesity-related vascular dysfunction in individuals with well-controlled diabetes. The analyses demonstrated that tirzepatide use significantly decreased CAVI accompanied by reduced SMI, BFI and glycemic parameters. A trend toward a decrease in UACR was also observed with tirzepatide, and the change in UACR showed a marginal positive correlation with ΔCAVI. BMI and CAVI tended to decrease to a greater extent in the subgroup receiving 10 or 15 mg of tirzepatide once-weekly compared to the subgroup receiving 5 or 7.5 mg. The efficacy of tirzepatide was unchanged irrespective of GLP-1 RA use before switching to tirzepatide. Baseline SMI, but not BFI, showed a negative correlation with ΔCAVI. The strength of the present study is to demonstrate that tirzepatide improves vascular function assessed by CAVI, and that this beneficial effect may be affected by body composition and improved kidney function. Tirzepatide is expected to improve kidney and vascular function in individuals with obesity complicated by type 2 diabetes who have adequate skeletal muscle mass.

In addition to improved glycemic control, several hypotheses may be proposed regarding the mechanism by which tirzepatide reduces CAVI. First, the results of this study suggest that tirzepatide may improve vascular function by modifying unfavorable body composition. We previously reported that CAVI decreases when excess visceral fat is reduced in individuals with obesity complicated by type 2 diabetes through a protein-sparing modified formula diet combined with exercise therapy [13]. In addition, non-surgical weight reduction therapy has been shown to improve systemic vascular function and, in parallel, exert favorable effects on kidney function [14]. Excessive visceral fat accumulation contributes to vascular toxicity through upregulation of oxidative stress pathways and chronic low-grade inflammation, both of which promote vascular dysfunction. Although the present study did not evaluate visceral fat accumulation, it is highly likely that tirzepatide-induced body composition improvement has a favorable effect on vascular function. On the other hand, greater CAVI improvement with tirzepatide was observed in individuals with higher baseline SMI. Incretin analogs, including semaglutide and tirzepatide, may induce sarcopenia by reducing skeletal muscle mass [15,16], and we previously found an association between a decrease in lean body mass and an increase in CAVI [17]. In this study, SMI decreased slightly after tirzepatide treatment, and baseline SMI was not associated with ΔSMI, suggesting that tirzepatide reduced SMI to a similar extent regardless of baseline SMI. Therefore, it is thought that tirzepatide is more likely to exert its vasoprotective effects in individuals with a relatively high baseline SMI. In contrast, in individuals with relatively low baseline SMI, excessive loss of skeletal muscle mass may occur, which could offset the beneficial vascular effects of tirzepatide. Notably, we have previously reported that the skeletal muscle–depleting effects of incretin analogs can be prevented by a protein-sparing modified formula diet [15]. Taken together, when prescribing tirzepatide, particularly at higher doses, therapeutic strategies that ensure adequate protein intake and incorporate exercise are recommended.

Second, kidney function improvement induced by tirzepatide may be related to changes in vascular function. This hypothesis is supported by the simultaneous reductions in both UACR and CAVI in tirzepatide treatment, as well as the positive correlation between these changes. CAVI has been shown to be closely related to kidney function in both cross-sectional [18] and longitudinal [19] studies, and UACR has also been associated with CAVI in individuals with diabetes [20]. Moreover, a longitudinal study reported that high baseline UACR independently predicts later development of new carotid plaques, suggesting that kidney damage may precede atherosclerosis progression [21]. Since CKD and systemic vascular dysfunction create a vicious cycle through pressure gradients, chronic inflammation and oxidative stress [22], it is plausible that tirzepatide could interrupt this cycle. Conversely, UACR has also been shown to decrease in individuals with severe obesity undergoing weight reduction therapies including bariatric surgery [23]. Therefore, further investigation is needed to determine whether the decrease in UACR after tirzepatide treatment reflects a pleiotropic kidney effect or a secondary effect of weight loss.

Finally, we hypothesize that the decrease in CAVI observed in this study is attributable to pleiotropic vascular effects of tirzepatide. Although tirzepatide tended to reduce both BMI and CAVI in a dose-dependent manner, changes in weight, SMI, BFI, and glycemic parameters did not correlate with ΔCAVI. These findings suggest that tirzepatide may lower CAVI independent of its weight-reducing and glucose-lowering effects. In a mouse model, tirzepatide mitigated aortic dissection by suppressing vascular inflammation and vascular smooth muscle contraction via inactivation of the NLRP3 inflammasome, a key regulator of inflammatory cytokines [24]. In addition, the pleiotropic vascular effects may include enhanced endothelial progenitor cell recruitment and increased nitric oxide production [25]. Thus, tirzepatide may exert protective effects on systemic and kidney vasculature even when obesity and hyperglycemia are not fully controlled.

In this study, ΔLDL-C and ΔTG also correlated with ΔCAVI, although these lipid parameters did not show significant changes with tirzepatide treatment. These findings should be interpreted as reflecting the physiological relationship between vascular function and lipid parameters, independent of the effects of tirzepatide. Since our previous study [26] has already reported that LDL-C and TG are independently associated with CAVI, the present results reconfirmed the relationship.

## 5 Limitations

The present study has several limitations. First, it was a single-center study with a small number of participants. Due to the small sample size, it may have been difficult to detect statistically significant differences. The observation period and the use of concomitant diabetes medications were not uniform. The observed association between tirzepatide administration and improved vascular function in this study does not establish causality, as the absence of a control group precludes definitive causal inference. Accordingly, these findings should be interpreted as hypothesis-generating. Additionally, since this study population primarily consisted of individuals with obesity and well-controlled diabetes, the findings may not be generalizable to individuals without obesity but have poorly controlled diabetes.

## 6 Conclusion

Tirzepatide may dose-dependently reduce body weight and CAVI, contributed by decreased UACR and relatively high baseline SMI. Although tirzepatide is expected to improve kidney and vascular dysfunction in individuals with obesity complicated by type 2 diabetes, careful attention to body composition is warranted.

## Takeaway clinical messages

• Treatment with tirzepatide significantly reduced CAVI, accompanied by decreases (or trends toward decreases) in SMI, BFI, UACR and glycemic parameters.
• The strength of the present study is the demonstration that tirzepatide improves vascular function, and that this beneficial effect may be affected by body composition and improved kidney function.
• Tirzepatide is expected to enhance kidney and vascular functions in individuals with obesity complicated by type 2 diabetes who maintain adequate skeletal muscle mass.

## Availability of data and materials

The data that support the findings of this study are not publicly available due to their containing information that could compromise the privacy of research participants, but are available from the corresponding author upon reasonable request. Further inquiries can be directed to the corresponding author.

## Statement of ethics

All procedures and data collection were in accordance with the ethical standards of the institutional and Japanese national research committees, and the ethical standards of the Helsinki Declaration of 1975. This study and consent procedure were reviewed and approved by the Ethics Committee of Toho University Sakura Medical Center (approval no.: S24045_S24018_S21023_S18061). Written informed consent was obtained from all participants prior to their inclusion in the study. All data were anonymized to protect participant privacy, and results are reported in aggregate form to prevent personal identification.

## Author contributions

Conceptualization, A.S.; Data acquisition, S.T., M.W. and Y.W.; Data curation and formal analysis, D.N.; Data interpretation, M.W., S.T., Y.W., M.I., Y.K., O.H., K.S. and A.S.; Writing—original draft preparation, D.N.; Writing—review and editing, M.W., S.T., Y.W., M.I., Y.K., O.H., K.S. and A.S. All authors have read and agreed to the published version of the manuscript.

## Declaration of generative AI and AI-assisted technologies

During the preparation of this work, the authors did not use any generative AI and AI-assisted technologies for purposes other than checking references.

## Sources of funding

Beyond payment to the research staff by the University, this research did not receive any specific grant from funding agencies in the publish, commercial, or not-for-profit sectors.

## Declaration of competing interests

Atsuhito Saiki has received lecture fees from Novo Nordisk Pharma Ltd., Eli Lilly Japan K.K., Tanabe Pharma Corporation, Kowa Company, Ltd., and Nippon Boehringer Ingelheim Co., Ltd. The other authors declare no conflicts of interest.
