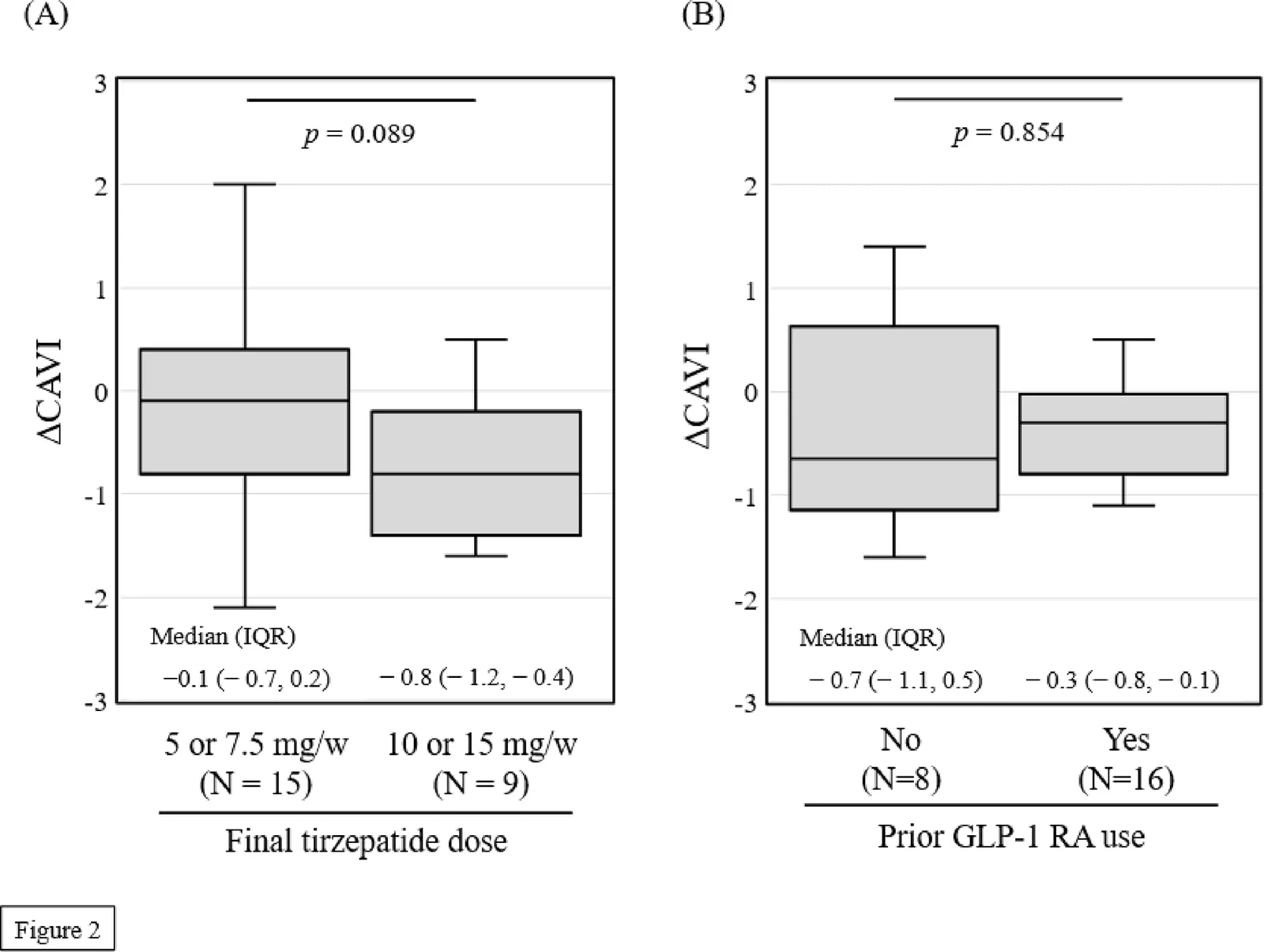# Corrigendum to “Impact of tirzepatide on systemic arterial stiffness assessed by cardio-ankle vascular index in individuals with obesity complicated by type 2 diabetes: A retrospective cohort study in Japan” [Obesity Pillars 19 (2026) 100291]

**DOI:** 10.1016/j.obpill.2026.100294

**Published:** 2026-07-02

**Authors:** Daiji Nagayama, Miwako Wakamatsu, Yasuhiro Watanabe, Maika Ikeda, Yuta Koshikawa, Osamu Horikawa, Sayaka Tsuji, Kohji Shirai, Atsuhito Saiki

**Affiliations:** a Department of Internal Medicine, Nagayama Clinic, Oyama-City, Tochigi, 323-0032, Japan; b Center of Diabetes, Endocrinology and Metabolism, Toho University, Sakura Medical Center, Sakura-City, Chiba, 285-0841, Japan; c Department of Internal Medicine, Mihama Hospital, Chiba-City, Chiba, 261-0013, Japan

The authors regret for errors in the display of several figures that were discovered after publication. Specifically, the **Graphical abstract**, Figs. 1 and 2 contained some garbled text.

The corrected versions of these figures shown below are identical to those attached to the manuscript submitted to *Obesity Pillars* and have not been modified in any way.

The authors assure readers that this error has not affected the rest of the manuscript or its conclusions. The authors apologize for any inconvenience caused.

**Figure:**
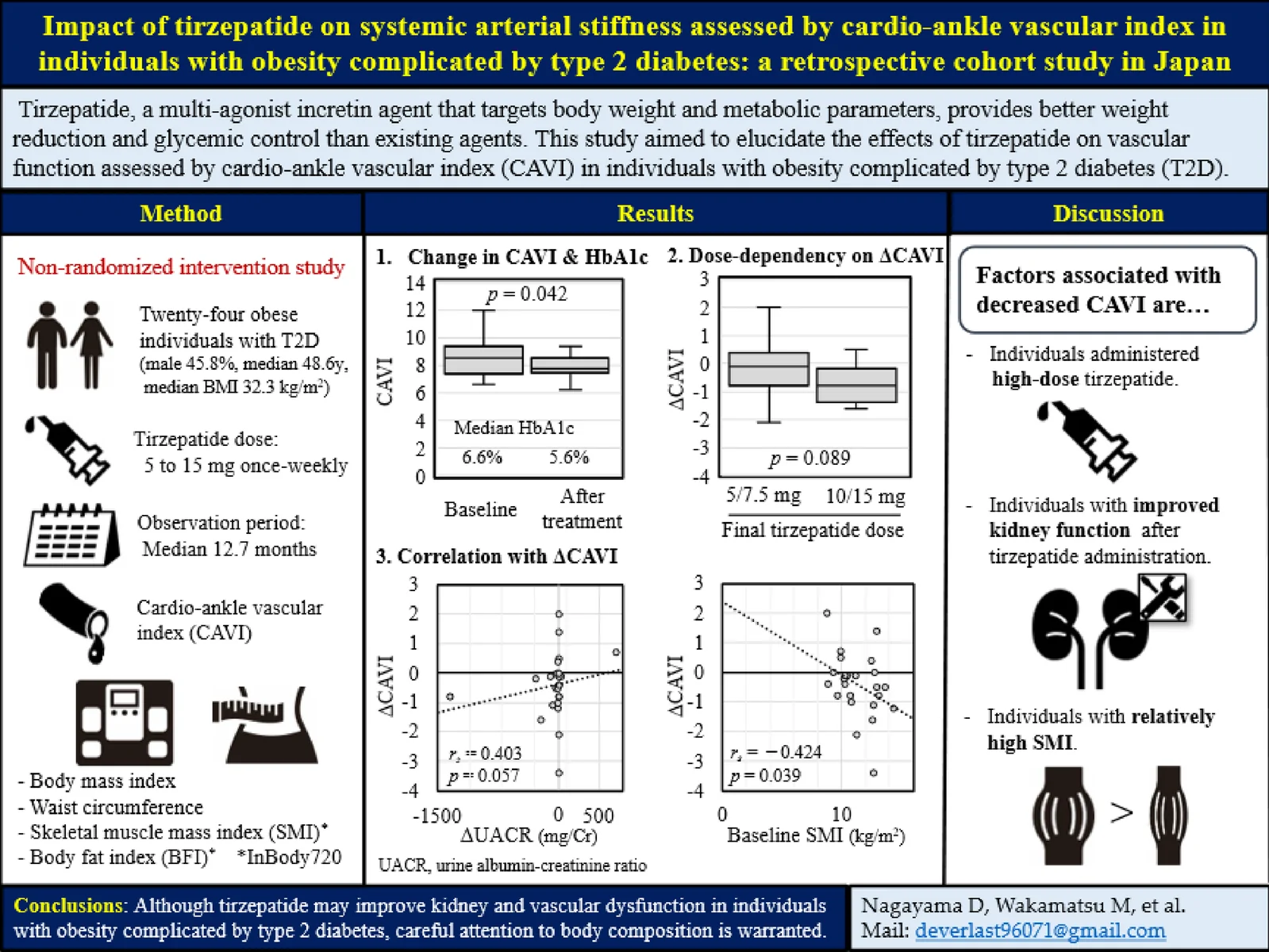

**Figure:**
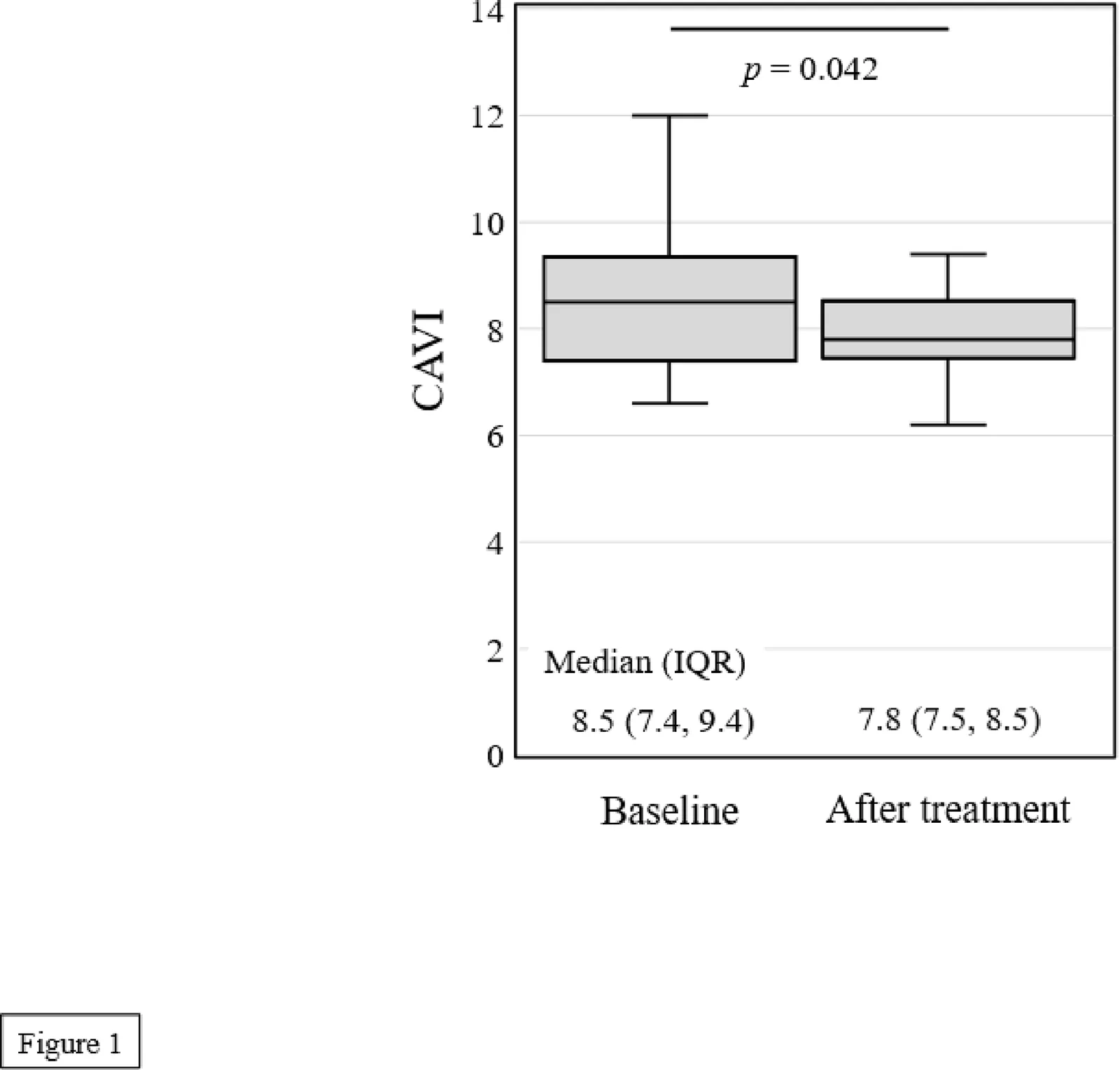

**Figure:**